# Polyvinyl Alcohol Assisted Iron–Zinc Nanocomposite for Enhanced Optimized Rapid Removal of Malachite Green Dye

**DOI:** 10.3390/nano13111747

**Published:** 2023-05-26

**Authors:** Muhammad Saad, Hajira Tahir, Seher Mustafa, Osama A. Attala, Waleed A. El-Saoud, Kamal A. Attia, Wessam M. Filfilan, Jahan Zeb

**Affiliations:** 1 Department of Chemistry, University of Karachi, Karachi 75270, Pakistan; saad@uok.edu.pk (M.S.); sehermustafa10@gmail.com (S.M.); 2 Department of Environmental and Health Research, The Custodian of the Holy Mosques Institute for Hajj and Umrah Research, Umm Al-Qura University, Makkah 21955, Saudi Arabia; oaahassan@uqu.edu.sa (O.A.A.); wekhamis@uqu.edu.sa (W.A.E.-S.); qurashisown@gmail.com (J.Z.); 3 Department of Biology, Al-Jammoum University College, Umm-Al Qura University, Makkah 24381, Saudi Arabia; kaattia@uqu.edu.sa; 4 Department of Biology, Aljumum University College, Umm Al-Qura University, Makkah 21955, Saudi Arabia; wmfilfilan@uqu.edu.sa

**Keywords:** adsorption, central composite design, isotherms, kinetics, nanocomposites, response surface methodology, thermodynamics, ultrasonic-assisted adsorption, wastewater treatment

## Abstract

Eliminating hazardous contaminants is a necessity for maintaining a healthy environment on Earth. This work used a sustainable method to create Iron–Zinc nanocomposites with polyvinyl alcohol assistance. Mentha Piperita (mint leaf) extract was used as a reductant in the green synthesis of bimetallic nanocomposites. Doping with Poly Vinyl Alcohol (PVA) caused a reduction in crystallite size and greater lattice parameters. XRD, FTIR, EDS, and SEM techniques were used to establish their surface morphology and structural characterization. The high-performance nanocomposites were used to remove malachite green (MG) dye using the ultrasonic adsorption technique. Adsorption experiments were designed by central composite design and optimized by response surface methodology. According to this study, 77.87% of the dye was removed at the optimum optimized parameters (10.0 mg L^−1^ was the concentration of MG dye at a time of 8.0 min, pH 9.0, and 0.02 g of adsorbent amount) with adsorption capacity up to 92.59 mg·g^−1^. The dye adsorption followed Freundlich’s isotherm model and the pseudo-second-order kinetic model. Thermodynamic analysis affirmed the spontaneous nature of adsorption due to negative ΔG^o^ values. As a result, the suggested approach offers a framework for creating an effective and affordable technique to remove the dye from a simulated wastewater system for environmental conservation.

## 1. Introduction

Nanomaterials have captivating interest largely due to their distinctive physicochemical characteristics, defined by their size, form, content, and crystallinity, which may all be determined. They are used in various fields, such as water treatment, biotechnology, catalysis, and medicine [1]. The metallic nanocomposites (MNCs), among the synthetic nanomaterials so far produced, have distinct characteristics such as catalysis, high chemical stability, electrical conductivity, and antibacterial properties [2]. On the basis of dimensions, nanocomposites are classified within four main categories, i.e., 3D (e.g., embedded network), 2D (e.g., nanoscale coatings), 1D (e.g., nanotubes), and 0D (e.g., embedded cluster). Classic methods for nanomaterials synthesis such as the chemical reduction method, polyol method, microemulsions, etc. are expensive, time-consuming, make use of toxic chemicals, and their application on a large scale is uneconomic; so, the usage of green strategies is becoming more popular due to their numerous advantages. The most commonly used reducing agents are polyphenols or glucose, which are extracted from plants and are used because of the manufacturing production of metallic and metal oxide nanomaterials. Likewise, mint is used to perform this experiment to improve the sustainability of developing the nanocomposites [3,4]. Caffeic acid, Rosmarinic acid, ferulic acid, and eugenol are the major phenolic contents present in mint [5].

Distribution, size, and shape are major variables that need to be considered while developing nanomaterials. Because of their high surface energy, nanomaterials have a tendency to aggregate, which reduces the available surface area for adsorption and lowers the overall effectiveness of the nanocomposites [6]. In order to develop a nanoparticle solution, a suitable medium or an extra surface preparation is frequently needed. Research is being conducted on a variety of organic and synthetic surface coatings, including kaolinite, bentonite, resin, ligands, starch, and synthetic polymers, to improve the effectiveness of metallic nanocomposites [7,8,9,10,11,12,13]. In the case of polymers, this matrix is often an organic matrix incorporating inorganic nanoparticles. Solid structures scattered at a nano size are thought to make up the structure of nanocomposites. Homopolymers or copolymers, neutral or charged, and hydrophilic or amphiphilic are some of the numerous categories of polymers used for the coating materials of metallic nanocomposites (MNCs). Hydrogen bonds and electrostatic pseudo-covalent bonding can be used to embed these polymers into the surface of MNCs. The outer type of the polymer-coated MNPs is often assumed to be hydrophilic, with good water stability and solubility.

Polyvinyl alcohol (PVA) has significant potential for groundbreaking innovations. It is a non-toxic, water-soluble, and biocompatible synthetic polymer and is mostly utilized in the pharmaceutical, packaging, and biomedical industries, with various purposes. The chemical structure of PVA is simple and consists of the main structure of carbons with hydroxyl groups. Because of intra- and intermolecular hydrogen interactions, PVA samples dissolve at high temperatures (about 80 °C) and require constant stirring [14]. Additionally, it has great film-forming properties, high chemical resistance, and a PVA surface layer that prevents nanocomposites from clumping together, which causes them to become stable and monodispersed [15,16,17]. An inventive method to create new materials with particular qualities has already been published, stabilizing the MNC’s surface using PVA [18,19,20]. Inorganic nanocomposites are often dispersed into polymer matrices using one of three methods. The filler is directly mixed with polymer matrices in solution in the first applications of the top-down method. The second relies on the in-situ production of inorganic nanoparticles with polymer. The third one entails the in-situ creation of both organic and inorganic materials. Bottom-up methods are used to describe the final two applications.

For human existence to continue on Earth, water is a necessary natural resource [21]. Unfortunately, unchecked population increase, fast industrialization, and protracted droughts have contaminated water supplies, posing major concerns to water sustainability [22]. The discharge of dyes and other toxins by the textile industry’s wastewater is a serious major source of water pollution [23]. These colors have a negative impact on both human health and the environment. The toxicity and carcinogenic properties among others are risks factor [24]. They pose a dangerous hazard to being removed from the effluent due to their limited biodegradability as well [23]. Malachite green (MG) is one such toxic dye. Moreover, in addition to its main function as a colorant, it also functions as an antimicrobial and an antibiotic. However, it is highly painful and poisonous if consumed, and it irritates and hurts the skin in the case of dermal exposure [25].

As the specific pollutant piles up at the surface of a non-toxic adsorbent, the creation of green adsorbents is important to remove various contaminants at a commercial level [26]. Additionally, among other wastewater treatment methods, adsorption is one of the most basic yet extremely efficient strategies for the removal of contaminants. By exposing the treatment assembly to ultrasonic waves, the speed of adsorption is increased during the ultrasonication process. Because of the convection, this increases the rate of mass transfer. Additionally, it stimulates the adsorbent surface that was created by physical phenomena [27]. The response surface method (RSM) of optimization is used to obtain the optimal responses, which are generated by the specified parameters. RSM is a category of design of experiments that assembles an experimental system using statistical and mathematical tools within a certain number of pre-planned trials to assess the impact of the experimental factors and identify the best responses. Evaluation of response variations in relation to the modification of the experimental conditions is also helpful [28]. RSM is the basis of CCD (central composite design), which is typically utilized for experiment development with the fewest number of experimental runs. The simultaneous evaluation of the interdependent factorial replies is appropriate, and the potential for data redundancy is eliminated [25].

To eradicate the unwanted MG dye from the effluent, nanocomposites were created utilizing a mint extract, which is a natural source. The current study emphasizes the sustainable growth of polymer-aided Fe-Zn nanocomposites, i.e., (Fe-Zn-PVA-NCs). The application of the RSM was used to optimize the trials. With two central points, the CCD was used to assess the association between the removal of MG and four variables (concentration of dye, amount of adsorbent, pH, and time). Different kinetics and isotherm models were used to show and explain equilibrium data. Studies on regeneration and desorption were also carried out.

## 2. Experimental

### 2.1. Materials and Methods

The following analytical grade chemicals were used: Zinc sulphate heptahydrate (ZnSO_4_·7H_2_O), Sodium hydroxide (NaOH), Sodium chloride (NaCl), Iron (II) sulphate heptahydrate (FeSO_4_·7H_2_O), Hydrochloric acid (HCl), and Ethanol.

**Figure 1:**
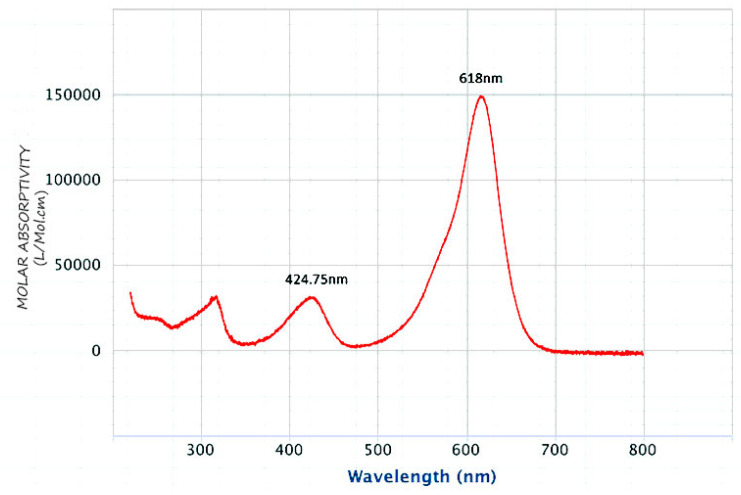
UV-Visible spectra of malachite green dye.

A pH meter (HANNA pH 211 microprocessor pH meter, Nusfalau, Romania) was used for pH measurements. The ultrasound-assisted adsorption procedure was carried out in an ultrasonicator (Elma-Hans Schmid Bauer Gmbh & Co, Singen, Germany) with a heating system. A UV–Vis spectrophotometer (T80, PG instruments LTD, Lutterworth, UK) at a wavelength of 618 nm, as shown in Figure 1, was used to measure the MG concentrations.

The MG dye solution of 500 mg L^−1^, as stock, was made in deionized water, which was then diluted to prepare the working standards.

Energy Dispersive X-ray Spectroscopy (EDS; Jeol Japan EX-54175 jMU, Tokyo, Japan) and Scanning Electron Microscopy (SEM; Jeol Japan JFC-1500) were used to study the composition and morphology of the Fe-Zn-PVA NCs, respectively, under an accelerated voltage of 15 kV. Fourier Transform Infrared Spectroscopy (FTIR; IR-Prestige 21-Schimadzu, Kyoto, Japan) was used to study the orientations and vibrations present and its chemical composites. The orientation and crystalline structure of the Fe-Zn-PVA NCs were analyzed by X-Ray Diffraction (XRD; Jeol, Japan, JDX 3532) using Cu Kα (λ = 1.54 Å).

### 2.2. Preparation of Mint Extract

The green extract was prepared using mint leaves (Mentha Piperita). About 20.0 g of fresh leaves of mint were purchased from a market in Karachi city. After cleaning, leaves were washed with deionized distilled water, air dried, cut into fine pieces, and later crushed using a mortar and pestle. The crushed leaves were added to 100 mL of deionized water. The process of extraction progressed further by heating this solution for 15 min at 353 K. After that, the contents were cooled and filtered. As a result, the extract that was recovered was employed as a reducing agent [29].

### 2.3. Preparation of Fe-Zn-PVA NCs

The nanoparticle of Zinc and Iron was produced independently, after the addition of ZnSO_4_·7H_2_O (0.1 M) and FeSO_4_·7H_2_O (0.1 M) at a volume ratio of 1:2 to the extract. At room temperature, the mixtures were swirled for 30 min. Bimetallic nanocomposites were created by combining the independently produced zinc and iron nanoparticles in a 1:1 *v*/*v* composition.

**Figure 2:**
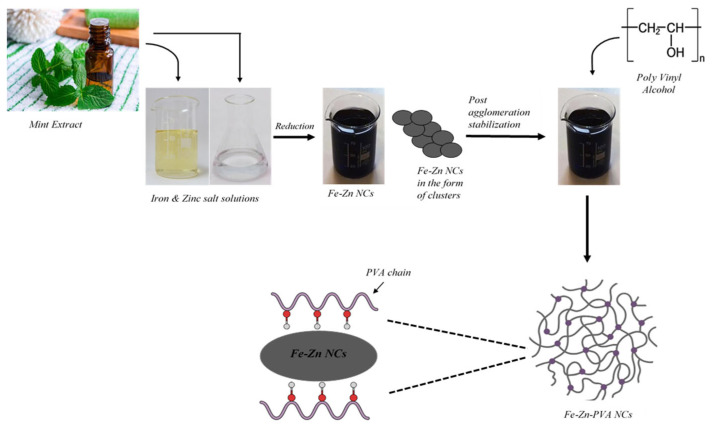
Synthesis pathway of Fe-Zn-PVA NCs.

For the preparation of the solution of PVA, about 10 g of PVA was added to 100 mL of deionized distilled water and stirred at a temperature of 80 °C until it was completely dissolved. The produced PVA solution was combined at a 1:1 proportion with the synthesized Fe-Zn nanocomposites, and the mixture was agitated for 30 min at room temperature. The synthesized nanocomposites were vigorously stirred with polymer solution for a particular time period for the completion of the reaction. The evaporation of the filtrate proceeded on a hot plate; after that, the Fe-Zn-PVA NCs were synthesized. They were then rinsed several times with ethanol and deionized water, and dried in an oven. The preparation scheme is shown in Figure 2 [30,31,32].

### 2.4. pH at Point of Zero Charge (pH_pzc_)

The pH value determined using the pH drift technique when the net charge of the nanocomposite surface becomes neutral is known as the Point of Zero Charges (pH_pzc_). We added specific amounts of NaOH and HCl; the pH of the starting set of samples of about 50 mL of NaCl (0.05 M) concentration was kept within 2–12. An appropriate quantity of Fe-Zn-PVA NCs was added to the respective system and pH of the solutions was measured after 24 h and 48 h intervals [24].

### 2.5. Ultrasonic-Assisted Adsorption Process

Adsorption of MG dye over the surface of Fe-Zn-PA NCs was accomplished in batch mode with the aid of the ultrasonication method. A solution with known concentrations of MG dye (5, 10, 15, 20, and 25 mg L^−1^) of around 50 mL was made. The solution’s pH was held steady at 9. Approximately, Fe-Zn-PVA NCs (0.02 g) were dissolved in the dye solution and ultrasonically processed (8.0 min) at room temperature after measuring the preliminary absorbance at 618 nm with a UV–Visible Spectrophotometer. The following equation was used to calculate the MG dye’s % removal [24].

(1) $$\%\;\mathrm{Removal}=\left(\frac{A_{i}-A_{f}}{A_{i}}\right)\times 100\%$$

where A_f_ and A_i_ show absorbance (final and initial), respectively. The adsorption capacity of nanocomposite (Fe-Zn-PVA) was computed as [24]

(2) $$q_{e}=\frac{\left(C_{o}-C_{e}\right)V}{W}$$

where C_e_ and C_o_ symbolize the equilibrium and initial dye’s concentration (mg L^−1^), respectively; V is the volume in L (L); and W shows the adsorbent mass in g (g).

### 2.6. Central Composite Design (CCD)

The most pertinent type of RSM design, central composite design (CCD), was used for optimization and modelling to impacts of 4 variables (independent)—sonication time (A), pH (B), Fe-Zn-PVA NCs quantity (C), and MG concentration—on the adsorption (with ultrasonic assistance) of dye (MG) against Fe-Zn-PVA NCs [27]. In order to remove the MG dye, the correlation between the four parameters described above and the two core areas was examined.

Analysis of Variance (ANOVA), response surface plots, and regression analysis were utilized for studying the effects of adsorption factors and determining the prerequisites for the highest possible adsorption efficiency [33].

### 2.7. Adsorption Kinetics, Isotherms, and Thermodynamics Studies

In order to conduct the adsorption kinetics investigation, 50 mL of the MG solution (10 mg L^−1^) was mixed with Fe-Zn-PVA NCs (0.02 g) and then sonicated for 1 to 10 min while the pH was maintained at 9.

Studies on thermodynamics and adsorption isotherm were conducted using optimum operating parameters (OOP) at different temperatures (308–323 K), i.e., MG dye (10 mg L^−1^) into dye solution (50 mL), Fe-Zn-PVA NCs (0.02 g), 9 pH, 8 min.

The process spontaneity was examined using thermodynamic studies. ∆S°, ∆G°, and ∆H° were evaluated by mapping ln K_D_ vs. T^−1^.

### 2.8. Surface Regeneration of Fe-Zn-PVA NCs

For four cycles, the capacity of Fe-Zn-PVA NCs to regenerate for the subsequent sorption of MG was examined. In the first sorption cycle, 0.02 g of Fe-Zn-PVA NCs were treated for 8.0 min in 50 mL MG dye with a concentration of 10 mg L^−1^ in 50 mL at pH 9.0. Following the first cycle’s effective dye sorption, Fe-Zn-PVA NCs were sorted and cleaned with distilled water. After drying, MG dye was removed from Fe-Zn-PVA NCs using a variety of solvents, including sulfuric acid (0.05 M), sodium hydroxide (0.05 M), hydrochloric acid (0.05 M), glacial acetic acid (0.05 M), and ethanol. Glacial acetic acid was the solvent used in all subsequent desorption tests since it was the most successful method for MG dye desorption. In the MG sorption and desorption investigations described above, the dye-desorbed Fe-Zn-PVA NCs were used again for the upcoming three cycles, and the sorption effectiveness of Fe-Zn-PVA NCs was noted [34].

## 3. Results and Discussion

### 3.1. Characterization of Fe-Zn-PVA NCs

**Figure 3:**
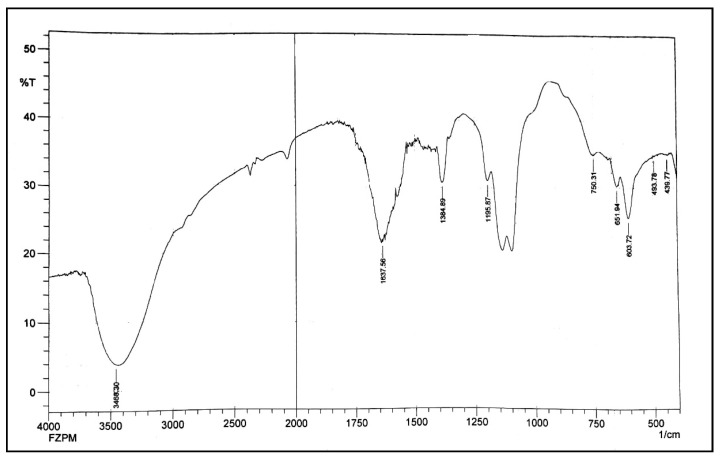
FTIR spectrum of Fe-Zn-PVA NCs.

Figure 3 shows the FTIR spectra of Fe-Zn-PVA nanocomposites to examine the bonding interactions and surface functionalization. The -OH of PVA molecules that are integrated onto Fe-Zn NCs are responsible for the wide peak with a center value of 3460 cm^−1^. Zn-O and Zn-Zn vibrations caused the vibrations of stretching to manifest at 439.77 cm^−1^, 493.78 cm^−1^, and a tiny height at 750 cm^−1^ [35]. Fe-O can be detected in the peaks at 603 cm^−1^ and 651 cm^−1^. Additionally, a large, irregular tip at 1637.56 cm^−1^ displays the mild twisting of the N-H group, whereas a tiny crest at 1384.89 cm^−1^ displays the curving of the (phenolic) OH group. Additionally, because of the significant broadening of the M-O-C bond, two peaks in the area of 1100 cm^−1^ namely, 1097.5 cm^−1^ and 1136.07 cm^−1^ can be observed [31,36].

**Figure 4:**
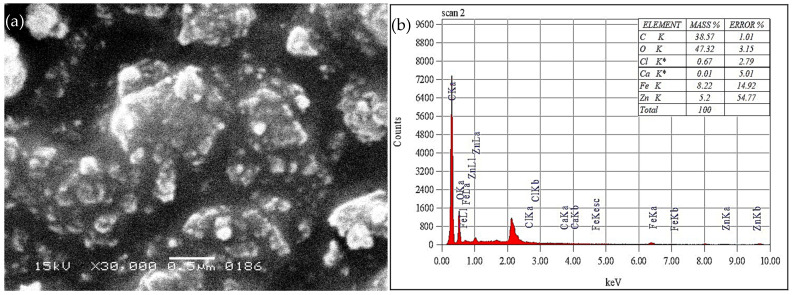
(**a**) SEM image of Fe-Zn-PVA NCs. (**b**) EDS spectrum of Fe-Zn-PVA NCs.

The SEM image of Fe-Zn-PVA NCs is shown in Figure 4a; the nanocomposites are between 80 to 100 nm in size and have spherical surfaces, which makes it easier for the dye to adhere to the Fe-Zn-PVA NCs.

The EDX (Energy Dispersive X-ray) spectrum, shown in Figure 4b, demonstrates the presence of carbon, oxygen, iron, and zinc in large numbers, but only small levels of calcium and chlorine, which may be present in the water utilized as interfering species [37].

**Figure 5:**
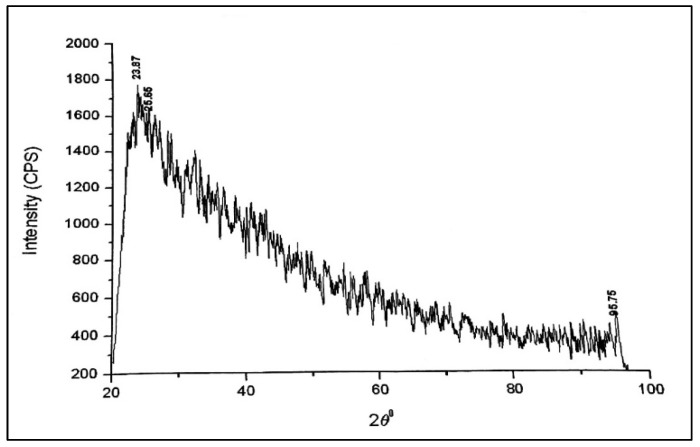
X-ray diffractogram of Fe-Zn-PVA NCs.

The XRD diffractogram displayed in Figure 5 shows that there were peaks at 23.87, 25.65, and 95.75, with miller indices of (100), (100), and (320) being detected at each peak, respectively. PVA’s interaction with Fe-Zn nanocomposites is what causes the decline in peak intensities and amalgamation of the diffraction peaks. Therefore, the XRD measurements show that the crystallite’s structural development is complicated [38].

### 3.2. Determination of Surface Neutrality by pH_pzc_ of Fe-Zn-PVA NCs

**Figure 6:**
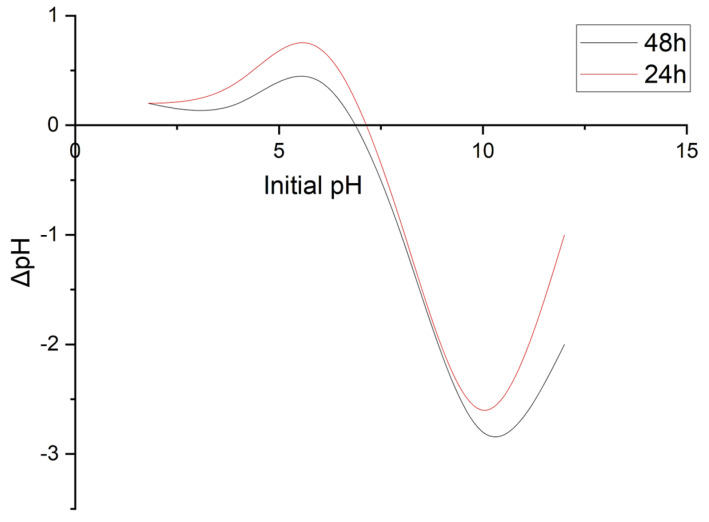
pH_pzc_ plot of Fe-Zn-PVA NCs.

The pH_pzc_ for Fe-Zn-PVA NCs was determined by producing a graph of ΔpH vs. initial pH, and it was determined to be 7.0, as shown in Figure 6.

**Figure 7:**
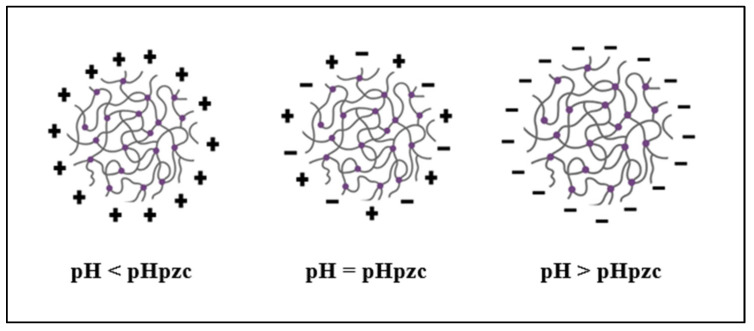
Surface charge of Fe-Zn-PVA NCs at different pH of solution.

As mentioned, if pH > pH_pzc_, the nanocomposites’ surface becomes negatively charged, attracting molecules with opposing charges and vice versa. Because Fe-Zn-PVA NCs are neutral at a pH value of 7.0 and MG is a dye with cationic characteristics with a surface positive charge, the two are attracted to one another. As the solution’s pH rises, the particle surface turns more negatively charged, leading to a larger percentage of dye removal, as shown in Figure 7 [33].

### 3.3. Design of Experiments

To ascertain the impact of the interactions between the key components for the eradication of MG, a four-factorial, five-level design was created, and the experimental runs were performed according to the central composite design provided in Table 1 along with the experimental and predicted responses.

Analysis Of Variance (ANOVA) was carried out to assess model significance along with the significance pertaining to all the variables and interactions. Table 2 shows that the model is considered statistically significant based on P and F values. The *p*-values of under 0.500 (i.e., 0.0001) and F-value (46.33) were found to indicate that the model is significant at a 95% confidence level. It is positive and demonstrates the strong predictability of the model, where the Lack of Fit F-value (F_tab_ = 4.74 > F_cal_ = 3.29) and *p*-value greater than 0.05 suggest the non-significance for the Lack of Fit that is desired [33].

It is clear from the ANOVA (shown in Table 2) that the most important factor for the MG removal is pH (*p* < 0.0001, SS = 2368.11, F = 501.14) and the next is time (*p* < 0.0001, SS = 155.14, F = 32.83). Another significant component is the interaction A^2^ (*p* < 0.0001, SS = 129.85, F = 27.48).

The statistics of the model summary are shown in Table 2. The great R^2^ (0.9774) demonstrates how well the data matched the fit regression line. The modified R^2^ of 0.9563 was a fair deal with the expected R^2^ of 0.8827, indicating that the regression line does not overfit the model, nor does the addition of an independent variable make the model worse [39,40].

**Table 2:** Analysis of Variance (ANOVA) for the adsorption of MG onto Fe-Zn-PVA NCs.

Source of Variation	Df ^a^	SS ^b^	MS ^c^	F-Value	*p*-Value
Model	14	3065	218.9	46.33	<0.0001
A	1	155.1	155.1	32.83	<0.0001
B	1	2368	2368	501.1	<0.0001
C	1	70.32	70.32	14.88	0.0016
D	1	48.56	48.56	10.28	0.0059
AB	1	2.45	2.45	0.5183	0.4826
AC	1	0.18	0.1806	0.0382	0.8476
AD	1	5.24	5.24	1.110	0.3088
BC	1	5.11	5.11	1.080	0.3150
BD	1	44.82	44.82	9.490	0.0076
CD	1	3.330	3.330	0.7048	0.4143
A²	1	129.8	129.8	27.48	<0.0001
B²	1	49.93	49.93	10.57	0.0054
C²	1	22.92	22.92	4.850	0.0437
D²	1	120.6	120.6	25.52	0.0001
Residual	15	70.88	4.730		
Lack of Fit	10	61.53	6.150	3.290	0.1004
Pure Error	5	9.350	1.870		
Cor Total	29	3136			

In the above table, ‘a’ denotes the degree of freedom, ‘b’ denotes the sum of squares, and ‘c’ denotes the mean squares of the datasets. The model summary for the removal of MG by synthesized nanocomposites revealed an exceptional model fitting as the R^2^ value was observed to be 0.9774 with an adjusted R^2^ value of 0.9563 and predicted R^2^ value of 0.8827.

The coded equation, as represented in Equation (3), is useful for identifying the comparative impact of the four factors (i.e., sonication time, pH, the concentration of MG, and amount of adsorbent) and their contact for MG removal.

(3) $$Y=65.86+2.54A+9.93B+1.71C-1.42D+0.3913\mathrm{AB}-0.1062\mathrm{AC}-0.5725\mathrm{AD}-0.5650\mathrm{BC}-1.67\mathrm{BD}\;+0.4563\mathrm{CD}-2.18A^{2}+1.35B^{2}+0.9142C^{2}+2.10D^{2}$$

This equation shows, because of the individual variables (sonication time, pH, amount of adsorbent and the concentration of pollutant), there has been a noticeable favorable effect for the elimination of MG dye (*p* < 0.05). Additionally, the reaction is inhibited by the dye concentration. Of the three factors, pH had the biggest impact on the MG removal from an aqueous solution.

### 3.4. Validation and Optimization of Fe-Zn-PVA NCs

The following factors were established to be the most efficient for removing MG dye: 8.0 min duration; pH 9.0; 0.02 g of the nanocomposite; MG dye concentration 10.0 mg L^−1^; and the highest removal of the model, which was anticipated to be 85.16%. The best conditions were verified by conducting a series of five tests at room temperature, and the observed results were 77.87% with a variance coefficient of 0.989%.

### 3.5. Effect of Parameters on MG Dye Removal

**Figure 8:**
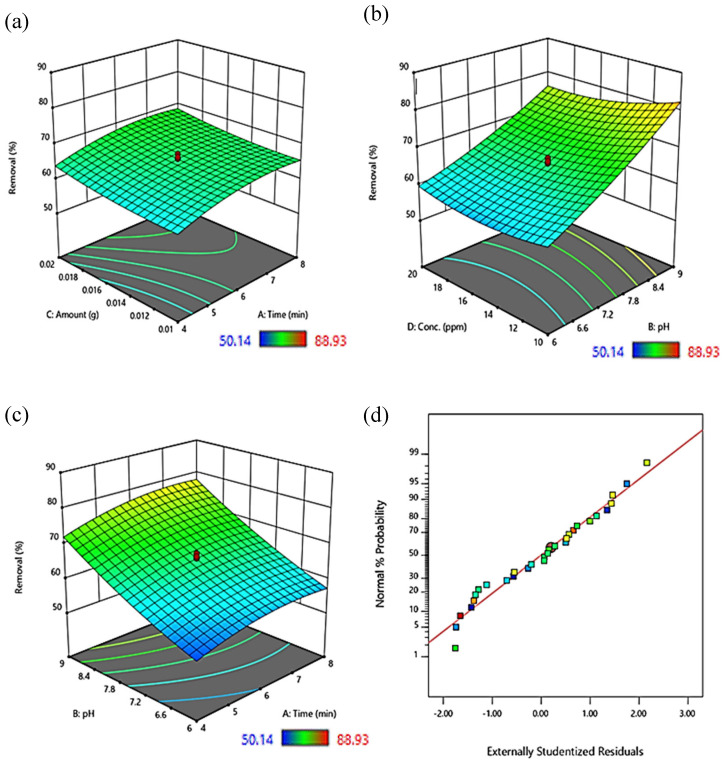
3D response surface plots showing % Removal as a function of (**a**) amount of adsorbent and sonication time, (**b**) concentration of dye and pH, (**c**) pH and sonication time, and (**d**) normal probability plot.

The 3D representations of surface graphs shown in Figure 8a–c illustrate the effects of four distinct factors on the responses: the quantity of adsorbent, time, pH, and adsorbate (dye) concentration.

The relationship between the volume of adsorbent and the duration of the ultrasonication is shown in Figure 8a. It was noted that as the adsorbent’s amount increased, the process efficiency grew due to the accessibility of more active sites, which increases the likelihood of adsorption. Additionally, the sonication period has a limited positive impact on the effectiveness of dye removal and might have negative impacts if it is prolonged due to system saturation and dye molecule desorption [37].

Adsorbate molecules are present at lower concentrations in the vicinity of accessible sites, and the percentage removal of adsorption is higher at lower than higher concentrations of adsorbate (MG), as seen in Figure 8b. As a result, the percentage of molecules that are adsorbed will be higher at lower concentrations than at higher concentrations. The reaction is inversely influenced by the solution’s pH. It was found that as pH rises, adsorption effectiveness rises as well. MG is a cationic dye, and when the pH > pH_pzc_, the adsorbent takes on a negative charge. As a result, electrostatic attraction causes a higher percent removal [33,37].

The reaction was observed to be at its optimum with a rise in pH and interaction duration, as illustrated in Figure 8c. A graph of the normal probability of residuals for the MG’s removal is shown in Figure 8d, and it represents that the actual points of data are evenly spaced out all over the center axis.

### 3.6. Adsorption Kinetics

**Figure 9:**
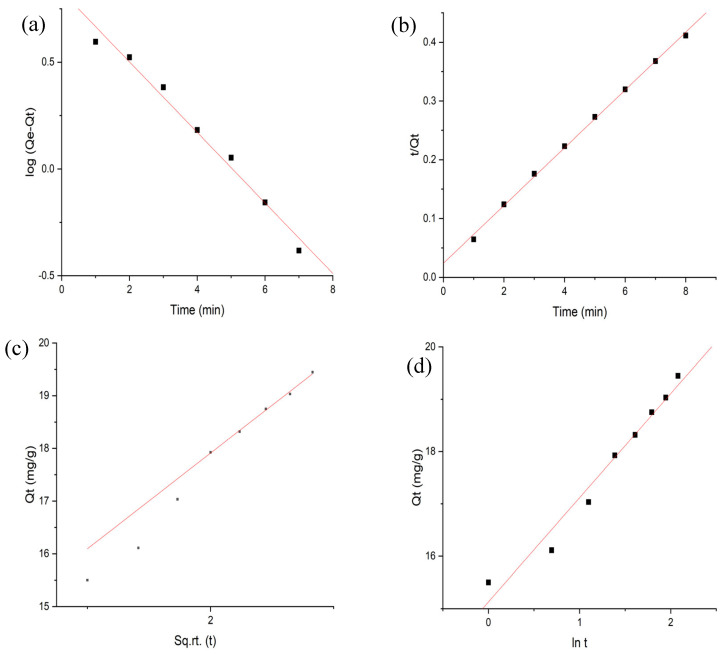
Kinetics plots of MG on Fe-Zn-PVA NCs: (**a**) pseudo-first-order model, (**b**) pseudo-second-order model, (**c**) intra-particle diffusion model, (**d**) Elovich model.

In Figure 9a–d, different models (Elovich model, intra-particle diffusion, pseudo-first-order, and pseudo-second-order) of kinetic studies were analyzed to observe the process mechanism. According to the results provided in Table 3, the pseudo-second-order was the model that best fit the data when compared to the other three models, with an R^2^ value of 0.9970. Furthermore, there was a fair agreement between the calculated and experimental Q_e_ values. However, even though the R^2^ value for the pseudo-first-order model was also high (0.983), there was a significant discrepancy between the estimated and observed Q_e_ values, suggesting that the process of adsorption does not closely follow the Lagergran equation. The intra-particle diffusion model and Weber and Morris’ equation were used to analyze the likelihood of intra-particle diffusion for the examination of a diffusion-controlled adsorption system. Figure 9c explains that the plot will not cross through the origins once extended back to the y-axis, indicating that boundary layer diffusion affects the adsorption. Additionally, a tiny curve in the straight line indicates that pore diffusion also occurred [41,42].

The Elovich model is used to analyze the processes involving chemical adsorption. It is also useful to define the extent of chemisorption. 

The values of respective constants are represented in Table 3. The R^2^ value shows that this study followed the respective model very well [27].

**Table 3:** Adsorption kinetics parameters for MG adsorption onto Fe-Zn-PVA NCs.

Models	Equations	Parameters	Values
Pseudo-first-order	$\log\left(Q_{e}-Q_{t}\right)=\log Q_{e}-\frac{k_{1}}{2.303t}$	Q_e_ (mg·g^−1^)	6.7842
		K_1_ (min^−1^)	−0.3802
		R^2^	0.983
Pseudo-second-order	$\frac{t}{Q_{t}}=\frac{1}{k_{2}Q_{e}^{2}}+\frac{t}{Q_{e}}$	Q_e_ (mg·g^−1^)	18.9393
		K_2_ (g·mg^−1^·min^−1^)	0.1858
		R^2^	0.9971
Intra-particle diffusion	$Q_{t}=k_{\mathrm{id}}t^{\frac{1}{2}}+C$	C (mg·g^−1^)	13.185
		K_id_ (mg·g^−1^)	2.2472
		R^2^	0.9883
Elovich Model	$Q_{t}=\frac{1}{\beta }\ln\left(\alpha \beta \right)+\left(\frac{1}{\beta }\ln\left(t\right)\right)$	α (mg·g^−1^·min^−1^)	3989
		β (g·mg^−1^)	0.5026
		R^2^	0.9707

Various parameters are involved in different models in the above table. Here, Q_e_, Q_t_, k_1_, k_2_, K_id_, and C denote the amount of MG adsorbed at equilibrium, amount of MG adsorbed at ultrasonicated time “t”, pseudo-first-order rate constant, pseudo-second-order rate constant, intra-particle diffusion rate constant, and thickness of the boundary layer, respectively.

### 3.7. Adsorption Isotherms

**Figure 10:**
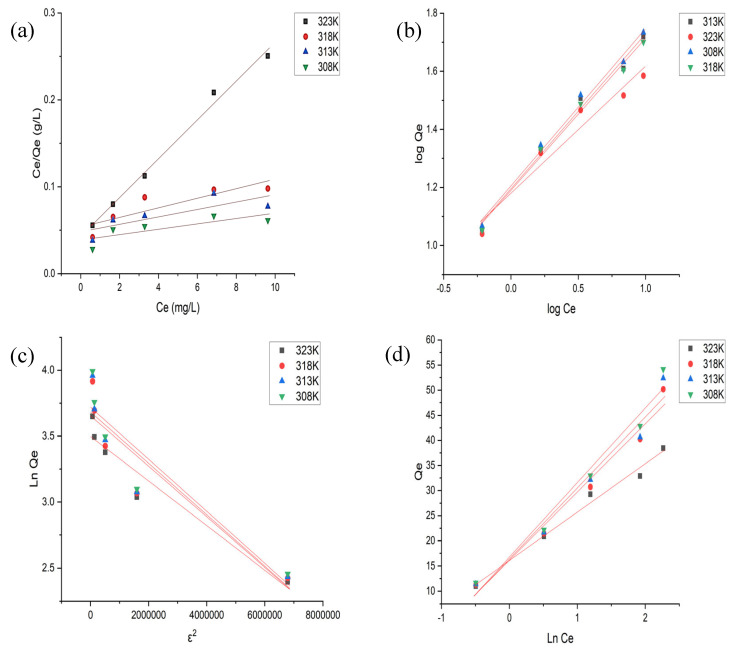
Isotherms plots of MG on Fe-Zn-PVA NCs: (**a**) Langmuir isotherm, (**b**) Freundlich isotherm, (**c**) Temkin isotherm, (**d**) D–R isotherm.

The adsorption equilibrium at various temperatures was ascertained using the Langmuir, Freundlich, Temkin, and D–R isotherm models, as shown in Figure 10a–d.

#### 3.7.1. Langmuir Isotherm Model

The monolayer adsorption that occurs on the homogeneous surface with limited identical adsorption sites is described by the Langmuir adsorption isotherm model [27,42].

The intercept (1/K_L_Q_m_) and the slope (1/Q_m_) of the graph were used to calculate the constant for the model parameters. The values of Q_m_ and K_L_ decreased as temperature rose, indicating that low temperatures are best for adsorbent and adsorbate interaction. The R_L_ values, which were observed to be below 1, suggested that the Langmuir model for the adsorption process was feasible [43]. It has been reported in the Literature [43] that if R_L_ > 1, the process is unfavorable; if R_L_ = 1, the process is linear; if 0 < R_L_ < 1, the process is favorable; and if R_L_ = 0, the process is irreversible.

The higher R^2^ values up to 0.9920 showed that the Langmuir isotherm model well-represented the adsorption equilibrium data.

#### 3.7.2. Freundlich Isotherm Model

To analyze the heterogeneity in the adsorbent surface, which results from the presence of several functional groups on the adsorbent surface, the Freundlich adsorption isotherm model is used [42].

The model’s K_F_ and n parameters were determined using the plot’s intercept (log K_F_) and slope (1/n), respectively, as presented in Table 4. Since n > 1, as documented in the Lature, the adsorption nature is considered to be favorable; “n” reflects the favorability of the adsorption, which was more than 1. The finding that the value of K_F_ decreases as temperature rises indicates that adsorption is more effective at decreased temperatures. The large values of R^2^ up to 0.9943 indicate that the Freundlich isotherm may be effectively applied to fit equilibrium data to MG dye adsorption onto the surface of Fe-Zn-PVA NCs [42].

#### 3.7.3. Temkin and Dubinin–Radushkevich Isotherm Models

The Temkin isotherm model is employed to study the effect of absorption heat, which changed during the adsorbate–adsorbate interactions. The constants of model parameters (K_T_ and B) were determined using the slope of the plot (B) and intercept (B ln K_T_), as illustrated in (Table 4). Additionally, the R^2^ > 0.8 indicates that this model is quite favorable.

The heterogeneous surface adsorption is described by the D–R isotherm model. Additionally, the energy of sorption, or E, predicts the kind of adsorption. When it is less than 8.00 kJ mol^−1^, physical adsorption is assumed to occur. Since, in Table 4, the E value is less than 8.00 kJ mol^−1^, it is clear that physical force has a greater influence on the current adsorption system [24,33].

**Table 4:** Adsorption isotherm parameters for MG adsorption onto Fe-Zn-PVA NCs.

Isotherm Models	Models’ Equations	Parameters	30 °C	35 °C	40 °C	45 °C
Langmuir	$\frac{C_{e}}{Q_{e}}=\frac{1}{Q_{m}K_{L}}+\frac{C_{e}}{Q_{m}}$ $R_{L}=\frac{1}{1+K_{L}C_{o}}$	Q_m_ (mg·g^−1^)	92.59	84.03	79.36	44.84
		K_L_ (L·mg^−1^)	0.3375	0.3028	0.2844	0.5198
		R_L_	0.2000	0.2170	0.7720	0.1240
		R^2^	0.8098	0.8408	0.8911	0.9920
Freundlich	${\log\;Q}_{e}={\log\;K}_{F}+\frac{1}{n}{\log\;C}_{e}$	n	2.721	1.545	1.611	2.285
		K_F_ (mg·g^−1^) (L·mg^−1^)^1/n^	40.48	19.09	17.57	15.15
		R^2^	0.9428	0.9820	0.9943	0.9521
Temkin	$Q_{e}={B\;\ln\;C}_{e}+{B\;\ln\;K}_{T}$	B (J·mol^−1^)	10.52	16.57	15.57	9.630
		K_t_ (L·g^−1^)	58.66	3.750	3.559	5.314
		R^2^	0.8951	0.8977	0.9197	0.9863
D–R	${\ln\;Q}_{e}={\ln\;Q}_{s}-{\beta \varepsilon }^{2}$ $\varepsilon =\mathrm{RT}\;\ln\;\left(1+\frac{1}{C_{e}}\right)$ $E=\frac{1}{\sqrt{2\beta }}$	Q_s_ (mg·g^−1^)	47.07	40.56	39.00	33.04
		β (mol^2^·kJ^−2^) (−1 × 10^−8^)	2.000	10.00	10.00	20.00
		ε	1550	1350	1270	1040
		E (kJ·mol^−1^)	5.000	2.240	2.240	1.580
		R^2^	0.9264	0.8466	0.8507	0.9328

In the above table, various isotherm parameters are represented by Q_m_, K_L_, K_F_, n, K_T_, B, R, T, β, Q_s_, ε, and E, which denote the amount of adsorbate per unit mass of adsorbent at equilibrium, Langmuir constant, Freundlich constant, constant related to adsorption capacity, Temkin isotherm equilibrium constant, Temkin constant related to heat adsorption, universal gas constant, absolute temperature, D–R isotherm constant, theoretical saturation capacity of the isotherm, D–R constant called Polanyi potential, and Mean free energy of sorption.

### 3.8. Adsorption Thermodynamics

To establish the temperature impact on the viability of MG dye adsorption onto Fe-Zn-PVA NCs, parameters of thermodynamics containing ΔG (free energy change), ΔS (entropy), and ΔH (enthalpy) were investigated [29]. The free energy change was calculated using the following equation. The change in free energy was estimated by the given equation:

(4) $$\Delta G=-{\mathrm{RTlnK}}_{D}$$

where R shows the universal gas constant (8.314 J mol^−1^ K^−1^), K_D_ shows the thermodynamic equilibrium constant, and T shows the temperature (K).

Moreover, in order to study the impact of temperature on the thermodynamic equilibrium constant, the equations used are given as follows:

(5) $${\mathrm{lnK}}_{D}=\frac{\Delta S}{R}-\frac{\Delta H}{\mathrm{RT}}$$

(6) $$\Delta G=-{\mathrm{RTlnK}}_{D}$$

**Figure 11:**
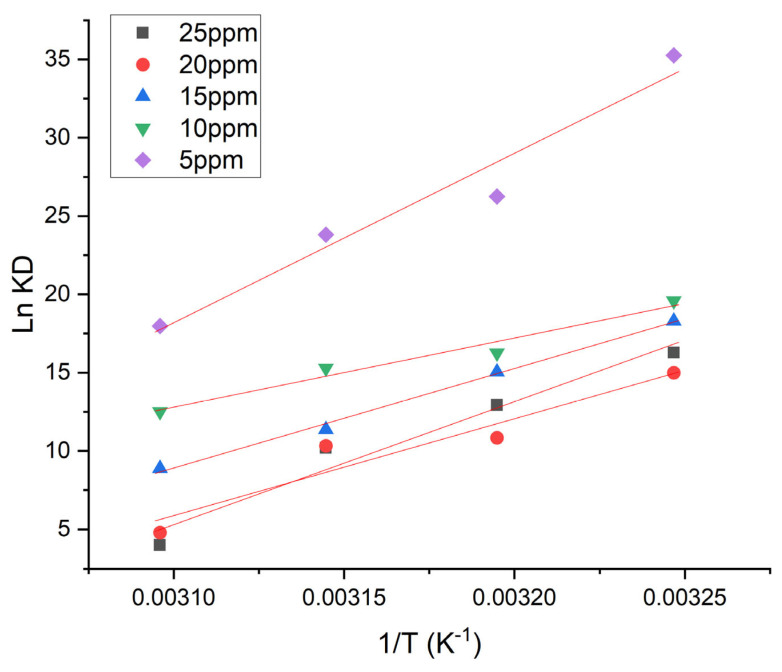
Thermodynamics studies of MG on Fe-Zn-PVA NCs.

K_D_, R, and T have the same representation as described in the above equation. Figure 11 shows the linear plot of ln K_D_ versus 1/T, and the values of intercept and slope were utilized to determine ΔS and ΔH, respectively. According to the values of ΔG and ΔH shown in (Table 5), the adsorption mechanism was exothermic and spontaneous, respectively.

### 3.9. Desorption and Surface Regeneration Studies

**Figure 12:**
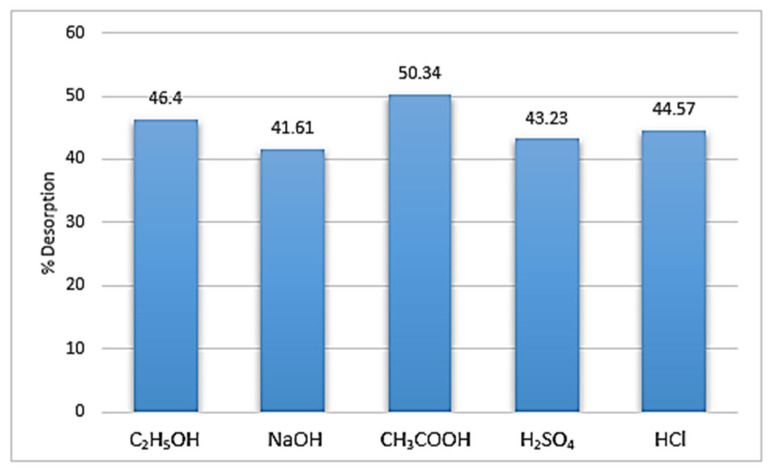
Desorption of Fe-Zn-PVA NCs.

The studies for desorption were carried out to meet the demand economically and to assess an adsorbent’s reusability. Figure 12 shows that, when compared to the other studied solvents, glacial acetic acid’s maximal desorption capability after 8 min of ultrasonication was observed to be 51.97%.

**Figure 13:**
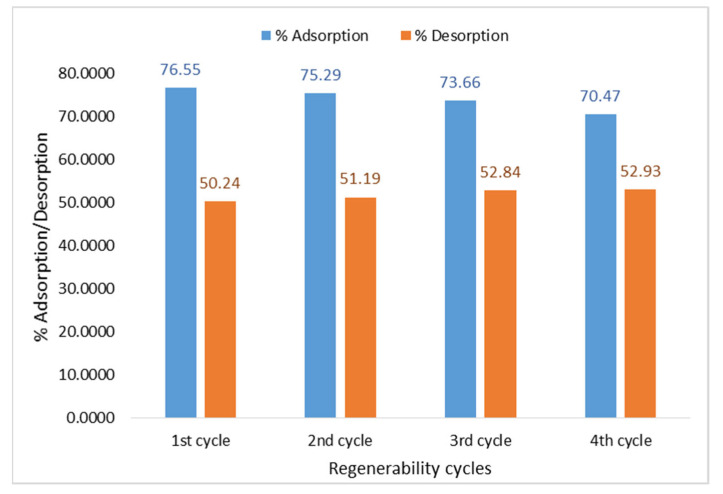
Regeneration using acetic acid as desorption agent.

The effectiveness of Fe-Zn-PVA NCs to adsorb MG was evaluated by conducting four cycles of the adsorption–desorption process in batch mode. MG sorption was discovered to be 76.55% in the first cycle, but after three cycles of subsequent adsorption and desorption, the % adsorption was reduced to 70.47, as shown in Figure 13. The strength of the binding forces that exist between the molecules of adsorbate and adsorbent determines the degree of reversibility of adsorption. Because of this, MG molecules that were adsorbed physically from Fe-Zn-PVA NCs departed the surface after 8 min but chemisorbed molecules found it difficult to do so, resulting in a higher percentage of adsorption than desorption, as seen in Figure 12 and Figure 13 [2].

**Figure 14:**
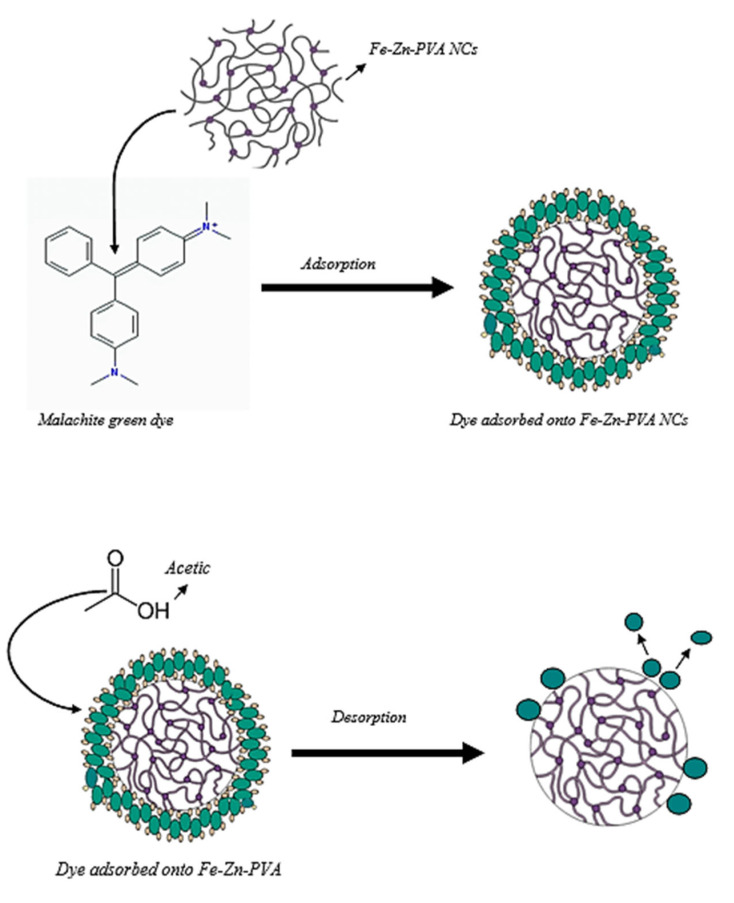
Adsorption and desorption pathway.

The mechanism of adsorption and desorption can be easily understood by the following scheme given in Figure 14.

### 3.10. Comparison with Reported Adsorbents

Table 6 presents the adsorption capacity for malachite green of various synthesized adsorbents with the Fe-Zn-PVA nanocomposites. It can easily be seen from the table that the nanocomposites were highly efficient in the adsorption of malachite green from the simulated wastewater.

## 4. Conclusions

In this study, green chemistry and nanotechnology were united in a manner by which Fe-Zn-PVA NCs were made using Mentha Piperita extract as a green source and then characterized using FTIR, SEM, EDS, and XRD. It was observed that the nanoparticles were homogeneous and between 80 and 100 nm in size. Response surface methodology was used to calculate the optimum operating parameters, i.e., 10.0 mg L^−1^ was the concentration of MG dye at a time of 8.0 min, pH 9.0, and 0.02 g of adsorbent amount, for the removal of malachite green dye. Under the optimum operational circumstances mentioned, the removal was discovered to be 77.87%. The Freundlich isotherm model and the pseudo-second-order model, respectively, were shown to be well matched to the equilibrium data by isotherm and kinetics analyses. Thermodynamic studies affirmed the spontaneity of the adsorption of MG dye over the surface of the green synthesized nanocomposites. A comparison with earlier research affirms that the nanocomposites are highly efficient in the adsorption of malachite green dye from aqueous medium. It has been determined that Fe-Zn-PVA NCs may be effectively used to remove dye from wastewater from the home and industrial sources.

## Figures and Tables

**Table 1:** Central composite design along with all the corresponding responses.

S. No.	Variables				%Removal of MG	
	Time (min)	pH	Amount of NP (g)	Pollutant Concentration (mg L^−1^)	Experimental	Predicted
1	8	6	0.020	10	62.01	62.30
2	6	4.5	0.015	15	53.23	51.39
3	8	6	0.020	20	61.02	62.57
4	6	7.5	0.015	15	67.34	65.86
5	6	7.5	0.015	5	79.04	77.10
6	6	7.5	0.005	15	64.34	66.10
7	10	7.5	0.015	15	63.82	62.25
8	6	7.5	0.015	15	63.27	65.86
9	4	9	0.010	10	78.75	76.77
10	8	9	0.010	10	84.94	84.00
11	6	10.5	0.015	15	88.93	91.13
12	4	9	0.020	20	76.95	74.23
13	8	9	0.020	10	83.29	85.17
14	6	7.5	0.015	15	65.99	65.86
15	6	7.5	0.015	15	66.00	65.86
16	6	7.5	0.025	15	74.34	72.94
17	6	7.5	0.015	25	69.10	71.41
18	4	6	0.020	20	58.62	59.63
19	4	9	0.020	10	77.58	78.37
20	4	9	0.010	20	71.04	70.81
21	4	6	0.010	20	56.25	53.95
22	8	6	0.010	20	58.04	57.32
23	6	7.5	0.015	15	66.14	65.86
24	2	7.5	0.015	15	50.14	52.08
25	8	9	0.020	20	79.49	78.74
26	6	7.5	0.015	15	66.44	65.86
27	8	9	0.010	20	76.56	75.75
28	8	6	0.010	10	56.58	58.87
29	4	6	0.010	10	52.40	53.21
30	4	6	0.020	10	56.68	57.07

**Table 5:** Thermodynamic parameters for MG adsorption onto Fe-Zn-PVA NCs.

Concentration	∆H	∆S	∆G			
(mg L^−1^)	(kJ mol^−1^K^−1^)	(J mol^−1^)	(KJ mol^−1^)			
			**308 K**	**313 K**	**318 K**	**323 K**
5	−35.029	−84.271	−9.123	−8.503	−8.382	−7.759
10	−23.194	−50.631	−7.618	−7.258	−7.211	−6.788
15	−40.419	−106.876	−7.443	−7.055	−6.427	−5.867
20	−57.116	−162.389	−6.935	−6.205	−6.175	−4.212
25	−73.275	−213.504	−7.143	−6.660	−6.139	−3.717

**Table 6:** Adsorption capacity of various adsorbents for the removal of malachite green.

Adsorbent	Adsorption Capacity (mg·g^−1^)	Reference
Fe-Zn-PVA nanocomposites	92.59	This research
Zn(OH)_2_-NP-AC	74.63	[44]
AgOH-AC nanoparticles	57.14	[45]
superparamagnetic sodium alginate coated Fe_3_O_4_ nanoparticles	47.84	[46]
Fe–Zn nanoparticles	21.74	[47]
Graphene Oxide–Gold Nanoparticles	6.164	[48]

## Data Availability

The manuscript is based on novel and original data, where the conclusion was determined through thorough experimentation. However, theoretical models and other pieces of information are available online and have been appropriately reported in the references.
